# Decorin Binding Proteins of *Borrelia burgdorferi* Promote Arthritis Development and Joint Specific Post-Treatment DNA Persistence in Mice

**DOI:** 10.1371/journal.pone.0121512

**Published:** 2015-03-27

**Authors:** Jemiina Salo, Annukka Jaatinen, Mirva Söderström, Matti K. Viljanen, Jukka Hytönen

**Affiliations:** 1 Department of Medical Microbiology and Immunology, University of Turku, Turku, Finland; 2 Turku Doctoral Programme of Biomedical Sciences, TuBS, Turku, Finland; 3 Turku Doctoral Programme of Molecular Medicine, University of Turku, Turku, Finland; 4 Department of Pathology, Turku University Hospital and University of Turku, Turku, Finland; University of Kentucky College of Medicine, UNITED STATES

## Abstract

Decorin binding proteins A and B (DbpA and B) of *Borrelia burgdorferi* are of critical importance for the virulence of the spirochete. The objective of the present study was to further clarify the contribution of DbpA and B to development of arthritis and persistence of *B*. *burgdorferi* after antibiotic treatment in a murine model of Lyme borreliosis. With that goal, mice were infected with *B*. *burgdorferi* strains expressing either DbpA or DbpB, or both DbpA and B, or with a strain lacking the adhesins. Arthritis development was monitored up to 15 weeks after infection, and bacterial persistence was studied after ceftriaxone and immunosuppressive treatments. Mice infected with the *B*. *burgdorferi* strain expressing both DbpA and B developed an early and prominent joint swelling. In contrast, while strains that expressed DbpA or B alone, or the strain that was DbpA and B deficient, were able to colonize mouse joints, they caused only negligible joint manifestations. Ceftriaxone treatment at two or six weeks of infection totally abolished joint swelling, and all ceftriaxone treated mice were *B*. *burgdorferi* culture negative. Antibiotic treated mice, which were immunosuppressed by anti-TNF-alpha, remained culture negative. Importantly, among ceftriaxone treated mice, *B*. *burgdorferi* DNA was detected by PCR uniformly in joint samples of mice infected with DbpA and B expressing bacteria, while this was not observed in mice infected with the DbpA and B deficient strain. In conclusion, these results show that both DbpA and B adhesins are crucial for early and prominent arthritis development in mice. Also, post-treatment borrelial DNA persistence appears to be dependent on the expression of DbpA and B on *B*. *burgdorferi* surface. Results of the immunosuppression studies suggest that the persisting material in the joints of antibiotic treated mice is DNA or DNA containing remnants rather than live bacteria.

## Introduction

Lyme borreliosis (LB), caused by spirochete *Borrelia burgdorferi* sensu lato, is a tick-borne infection common in northern hemisphere [1]. *Borrelia burgdorferi* sensu lato is further classified into several genospecies of which *Borrelia burgdorferi* sensu stricto (*B*. *burgdorferi*), *Borrelia garinii* and *Borrelia afzelii* are clinically the most important human pathogens. From the inoculation site in the skin, the bacteria can disseminate to other organs like the heart, joints and the nervous system, and cause a persistent infection in these foci. However, the molecules targeting the spirochete to the various tissues remain incompletely characterized.

Animal models, especially with mice, are widely used to study the dissemination and treatment response of LB. *B*. *burgdorferi* infected C3H mice develop prominent joint manifestations with joint swelling, periarticular oedema and leukocyte infiltration [2]. Some studies suggest that *B*. *burgdorferi* infected and antibiotic treated mice still harbour live but obviously attenuated and non-cultivable bacteria especially in collagen-rich tissues [3–5]. Parallel results have been obtained also using dogs and rhesus macaques [6, 7]. We have previously shown that a part of *B*. *burgdorferi* infected C3H/He mice treated with ceftriaxone became *B*. *burgdorferi* culture positive after immunosuppression by anti-TNF-alpha [8]. In contrast, recent mouse data obtained using intravital microscopy of antibiotic treated mice suggest that persisting *B*. *burgdorferi* antigens rather than an on-going infection can be detected in mouse joint after the treatment [9]. Taken together, the above animal data suggest the persistence of some form of *B*. *burgdorferi* remnants in animal joints after antibiotic treatment. The molecular mechanisms of this phenomenon are poorly understood.

*Borrelia burgdorferi* sensu lato has several adhesins, which are of critical importance for the virulence [10, 11]. Two such adhesins are decorin binding proteins A and B (DbpA/B) [12–16]. DbpA/B mediate bacterial attachment to decorin, which is a major component at the extracellular matrix, especially in the joint, skin and endothelial tissue [17–19]. The importance of *B*. *burgdorferi* adhesion to decorin is further underlined by the partial resistance of decorin deficient mice to LB [20]. Recent mouse data suggest a role for DbpA in the development of joint manifestations in mice [21, 22]. Interestingly, it is suggested that decorin rich tissues, like the joint, might serve as a protective niche for *B*. *burgdorferi* where the bacteria can hide from the immune response [23].

The present study was undertaken to test the hypothesis that DbpA and/or B are required for the development of joint manifestations and for post-treatment persistence of *B*. *burgdorferi* in mice. The results demonstrate that, indeed, the infection of mice with a *B*. *burgdorferi* strain that expresses both DbpA and B adhesins enables such progression of the infection that leads to arthritis development and post-treatment persistence. Results of our immunosuppression experiments suggest that the persisting material in the joints of mice infected with DbpA and B expressing bacteria and treated with ceftriaxone is DNA or DNA containing remnants rather than live bacteria.

## Materials and Methods

### *B*. *burgdorferi* strains

The study was conducted using previously characterized *B*. *burgdorferi* strains [16]. *dbpAB* knock out strain, Δ*dbpAB*/E22/1 (Δ*dbpAB*), the DbpA and B expressing strain, Δ*dbpAB*/*dbpAB*/2 (Δ*dbpAB*/*dbpAB*), the DbpA expressing strain, Δd*bpAB*/*dbpA*/1 (Δ*dbpAB*/*dbpA*), and the DbpB expressing strain, Δ*dbpAB*/*dbpB*/1 (Δ*dbpAB*/*dbpB*) in *B*. *burgdorferi* B31 5A13 background are identical in all other aspects of their genetic composition but differ in the ability to express DbpA and/or B. The spirochetes were cultivated in Barbour-Stoenner-Kelly II (BSK II) medium containing kanamycin (200 μg/ml, Sigma-Aldrich, St. Louis, MO, USA) and gentamycin (50 μg/ml, Biological Industries, Beit-Haemek, Israel) at 33°C. The minimal inhibitory concentration (MIC) of ceftriaxone was determined by culturing Δ*dbpAB*/*dbpAB* and Δ*dbpAB* in two-fold dilutions of the antibiotic in BSK II medium covering a concentration range of 0.5–0.002 μg/ml. Dark-field microscopy was used to detect the growth of the bacteria.

### Ethics Statement

This study was carried out in strict accordance with the recommendations in the Finnish Act on the Use of Animals for Experimental Purposes of Ministry of Agriculture and Forestry in Finland. The protocol was approved by the National Animal Experiment Board in Finland (permission number STH619A). All efforts were done to minimize suffering of the animals.

### Experimental design

Four weeks old female C3H/HeNhsd (C3H/He) mice (Harlan, Netherlands) were infected with 10^6^ Δ*dbpAB*/*dbpAB* (40 mice), Δ*dbpAB*/*dbpA* (8 mice), Δ*dbpAB*/*dbpB* (8 mice) or Δ*dbpAB* (38 mice) bacteria by intradermal syringe inoculation in the lower back. Twelve control animals were injected with an equal volume of PBS.

**Fig 1:**
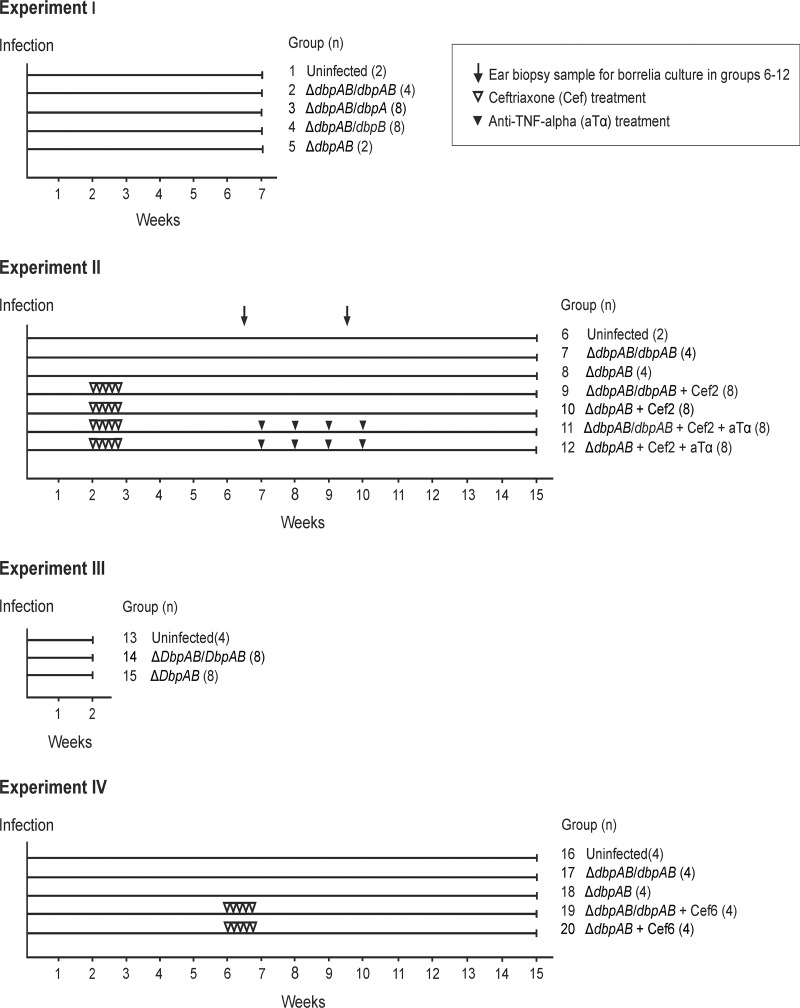
Design of the mouse experiments. In Experiment I, four Δ*dbpAB*/*dbpAB* (group 2), eight Δ*dbpAB*/*dbpA* (group 3), eight Δ*dbpAB*/*dbpB* (group 4), two Δ*dbpAB* (group 5) infected animals and two uninfected control animals (group 1) were killed at seven weeks of infection. In Experiment II, 16 infected animals (groups 4 and 5) were treated with ceftriaxone and 16 (groups 6 and 7) with ceftriaxone and anti-TNF-alpha. The ceftriaxone treatment was started at two weeks (25 mg/kg twice a day for 5 days) and the anti-TNF-alpha treatment at seven weeks of infection (10 mg/kg once a week for 4 weeks). Ear biopsy samples were collected at 6 and 9 weeks of infection to monitor the dissemination of the infection. In Experiment III, mice were killed at two weeks to study infection kinetics and bacterial load in joints. In Experiment IV, eight infected animals were treated with ceftriaxone at six weeks of infection (groups 14 and 15).

In experiment I (Fig. 1), four animals were infected with Δ*dbpAB*/*dbpAB* (group 2), eight with Δ*dbpAB*/*dbpA* (group 3), eight with Δ*dbpAB*/*dbpB* (group 4), and two with Δ*dbpAB* (group 5). Two uninfected animals (group 1) were negative controls. The development of joint manifestations was monitored by measuring the medio-lateral diameter of the hind tibiotarsal joints once a week. The measurer was blinded to the group’s identity. The mice were killed at seven weeks of infection. Tissue samples from ear, bladder and hind tibiotarsal joint were collected for culture.

In experiment II, 20 animals were infected with Δ*dbpAB*/*dbpAB* (groups 7, 9 and 11) and 20 animals with Δ*dbpAB* (groups 8, 10 and 12). Two uninfected animals (group 6) were negative controls. Sixteen animals (groups 9 and 10) were treated with ceftriaxone and 16 animals (groups 11 and 12) with ceftriaxone and anti-TNF-alpha. The ceftriaxone treatment was started at two weeks and the anti-TNF-alpha treatment at seven weeks of infection. Ceftriaxone (Rocephalin®, Roche, Mannheim, Germany) was administered twice a day 25 mg/kg intraperitoneally for five days. Rat murine chimeric TNF-alpha antibody of IgG2ak isotype (Centocor, Malvern, PA, USA) was administered once a week 10 mg/kg intraperitoneally for four weeks. The development of joint manifestations was monitored as described above. The mice were killed at 15 weeks of infection. Tissue samples from ear, bladder and hind tibiotarsal joint were collected for culture and PCR analyses. Blood was collected for serology, and one tibiotarsal joint for histology.

In experiment III, eight Δ*dbpAB*/*dbpAB* (group 14), eight Δ*dbpAB* (group 15) infected animals, and four uninfected control (group 13) animals were killed at two weeks of infection. Samples from ear, bladder and hind tibiotarsal joint were collected for culture. One hind tibiotarsal joint was collected for PCR analysis of *B*. *burgdorferi* tissue load, and blood was collected for serology.

In experiment IV, eight animals we infected with Δ*dbpAB*/*dbpAB* (groups 17 and 19) and eight animals with Δ*dbpAB* (groups 18 and 20). Four uninfected animals (group 16) were negative controls. Eight animals (groups 19 and 20) were treated with ceftriaxone at six weeks. The development of joint manifestations was monitored as explained above. The mice were killed at 15 weeks of infection. Tissue samples from ear, bladder and hind tibiotarsal joint were collected for culture and PCR analyses. Blood was collected for serology.

### Preparation and *B*. *burgdorferi* culture of tissue samples

In experiments II, the infection status of the mice was assessed by culturing ear biopsy samples at 6 and 9 weeks of infection. Ear, bladder and hind tibiotarsal joint samples were collected at seven weeks (experiments I), at 15 weeks (experiments II and IV), or at 2 weeks (experiment III) of the infection. All instruments were disinfected in ethanol between the dissections of the different samples. The tissue samples were grown in BSK II medium supplemented with phosphomycin (50 μg/ml; Sigma-Aldrich) and rifampin (100 μg/ml; Sigma-Aldrich) at 33°C for a maximum of 6 weeks.

### DNA extraction and PCR analysis

Ear, bladder and joint tissue samples were stored at -20°C before the DNA extraction. Tissue samples were incubated with proteinase-K (275 μg/ml, Promega, Madison, WI, USA) at 56°C for overnight before the DNA was extracted using NucliSENS easyMAG kit (Biomérieux, Marcy l'Etoile, France) according to manufacturer's instructions.

PCR analyses were performed using two different methods. All runs included a positive and negative control. A nested PCR was performed using two sets of primers targeting the chromosomal flagellin gene (*flaB*) according to the method described previously [24]. The outer primers were designed to amplify a 437 base pair fragment, and the inner primers a 277 base pair fragment of the gene. The PCR products were analysed on agarose gels.

Real-time PCR was performed using LightCycler 480 Probes master kit and LightCycler 480 II equipment (Roche). A 102 base pair product of *ospA* gene was amplified according to the method described by Ivacic and co-workers [25]. The minimal sensitivity of PCR was 40 bacterial cells. The *ospA* PCR was run quantitatively of the joint samples with 100 ng of extracted DNA as template and calculating the actual bacterial load with a standard curve. Data are expressed as the number of *B*. *burgdorferi* genomes per 100 ng of extracted DNA. The quantitative PCR was repeated three times.

### Serology

Whole *B*. *burgdorferi* antigen, C6 peptide, and DbpA and DbpB specific IgG antibodies were measured using in house enzyme immunoassays. *B*. *burgdorferi* B31 (ATCC 35210) whole cell lysate, biotinylated C6 peptide (Biotin-MKKDDQIAAAIALRGMAKDGKFAVK) or recombinant DbpA or DbpB of *B*. *burgdorferi* [26] were used as antigens.

Microtiter plates (Thermo Fisher Scientific, Vantaa, Finland) were coated with *B*. *burgdorferi* lysate (20 μg/ml), or DbpA or DbpB (10 μg/ml) in PBS, and washed three times with washing solution (H2O, 0.05% Tween 20, Merck, Hohenbrunn, Germany). Serum sample was diluted 1:100 to 1% bovine serum albumin (BSA, Serological Proteins Inc., Kankakee, IL, USA) in PBS. The wells were incubated with the diluted serum, washed as above, and incubated with PBS diluted goat anti-mouse HRP-conjugated IgG antibody (1:8000, Santa Cruz Biotechnology, Santa Cruz, CA, USA, SC-2031, Lot #I2513). After washings, ortho-phenylene-diamine (OPD, Kem-En-Tec Diagnostics A/S, Taastrup, Denmark) was added for 15–30 min before the reaction was stopped with 0.5 M H_2_SO_4_ and absorbances (OD_492_) were measured with Multiskan EX spectrophotometer (Thermo Fisher Scientific). All incubations were at 37°C for 1 hour, except for the substrate. Results are expressed as OD_492_ values and all samples were analysed in duplicate.

The measurement of C6 peptide specific antibodies was performed as above with the following exceptions: C6 peptide in PBS (5 μg/ml) was coated on streptavidin precoated plates (Thermo Fisher Scientific), the plates were saturated with 1% normal sheep serum-PBS (NSS-PBS), and mouse sera and secondary antibody were diluted in NSS-PBS.

### Histology

One tibiotarsal joint of each mouse (experiment II, groups 6–12) was formalin-fixed, demineralized, embedded in paraffin, sectioned at 5 μm, and stained with hematoxyline-eosin (HE) using routine histology techniques. Findings of joint disease were evaluated in sagittal joint sections by an experienced pathologist (MS) blinded to the experimental protocol.

### Statistical analysis

Statistical analyses of joint diameter, serum antibody levels and bacterial load in joint samples, were performed with analysis of variance (ANOVA, IBM SPSS Statistics 22) when there were more than two groups. Statistical analysis of the bacterial load in Experiment III was done using independent samples T-test. In the joint diameter measurements, animal-specific means were used as independent observations. Statistical significance was determined as p≤0.05. When comparing *B*. *burgdorferi* infected mice to non-infected controls, Bonferroni correction was used. In the serum antibody and bacterial load analysis Post Hoc comparisons between means were done with Dunnett *t* test when there was a clear control, otherwise Tukey’s honestly significant difference test was used.

## Results

### Arthritis development in Δ*dbpAB*/*dbpAB*, Δ*dbpAB*/*dbpA*, Δ*dbpAB*/*dbpB* and Δ*dbpAB* infected mice

**Fig 2:**
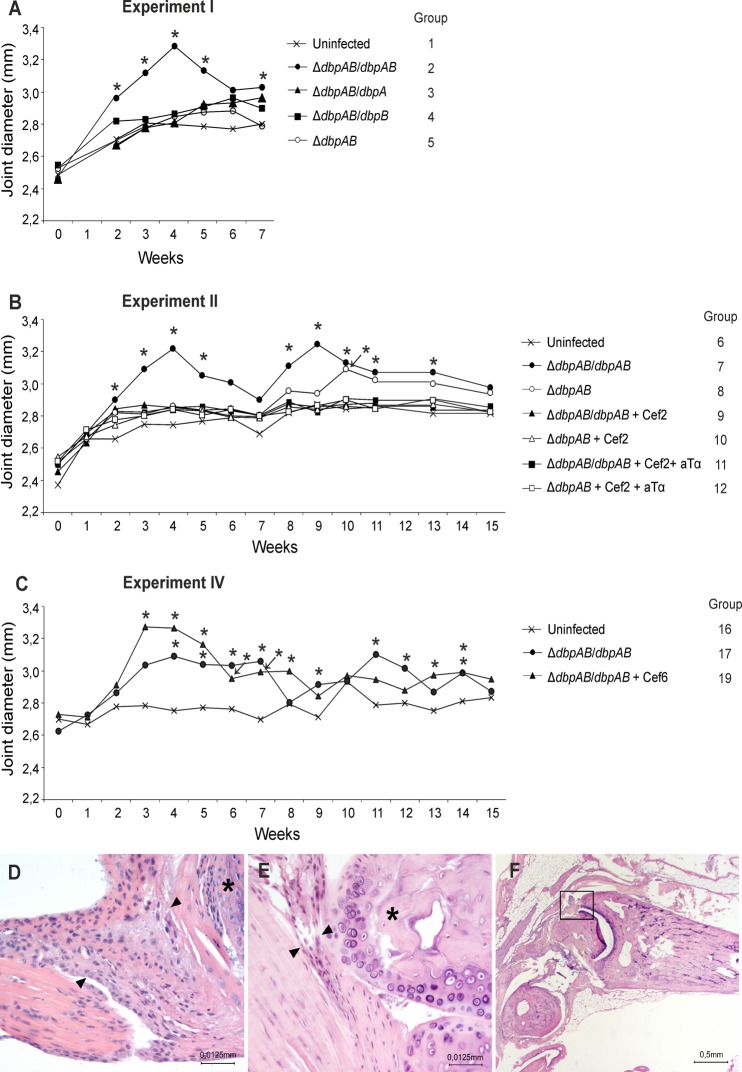
Joint swelling and histology. In experiment I (A), II (B) and IV (C), the development of joint swelling was monitored by measuring the medio-lateral diameter of the hind tibiotarsal joints once a week. Each curve represents the mean of the study group. Asterisks denote significant difference from uninfected mice (P ≤ 0.05). Paraffin-embedded tissue sections were prepared from Δ*dbpAB*/*dbpAB* (D; group 7) and Δ*dbpAB* (E; group 8) infected and uninfected control (F; group 6) mice at 15 weeks of infection and stained for HE. Arrowheads indicate the synovial membrane, and asterisks indicate articular cartilage surface in panels D and E. The original magnification in panels D and E is ×400 and in panel *F* ×20. Panel F indicates the anatomical structure shown in panels D and E. “Cef” Ceftriaxone treatment, “aTα” anti-TNF-alpha treatment.

An initial analysis (Experiment I) of the development of joint manifestations in mice infected the three different *B*. *burgdorferi* strains expressing either DbpA, DbpB or both DbpA and B, or with the strain lacking DbpA and B expression was performed. The joint diameter graph shows that Δ*dbpAB*/*dbpAB* is the only strain that causes a clear and prominent joint swelling with a peak at four weeks (Fig. 2A, group 2). All mice were *B*. *burgdorferi* culture positive in at least two of the three collected tissue samples (ear, bladder, joint) at seven weeks of infection (Table 1). These results show that all studied strains cause a disseminated infection in mice, but only the strain expressing both DbpA and B cause joint manifestations. Thus, for the further studies of arthritis development and post-treatment persistence, Δ*dbpAB*/*dbpAB* and Δ*dbpAB* were selected.

**Table 1:** *B*. *burgdorferi* culture results of Experiment I at seven weeks of infection.

		Culture at 7 wk		
Group	Strain/treatment	Ear	Bladder	Joint
1	Uninfected	0/2	0/2	0/2
2	Δ*dbpAB*/*dbpAB*	4/4	4/4	4/4
3	Δ*dbpAB*/*dbpA*	8/8	7/7^a^	7/8
4	Δ*dbpAB*/*dbpB*	5/8	8/8	8/8
5	Δ*dbpAB*	0/2	2/2	2/2

a One sample not available for culture

### Long-term follow-up of arthritis in Δ*dbpAB*/*dbpAB* and Δ*dbpAB* infected mice

In Experiment II, weekly joint diameter measurements were continued until week 15. The joint diameter graph shows that Δ*dbpAB*/*dbpAB* caused an evident joint swelling now with two statistically significant (P ≤ 0.05) peaks at 4 and 9 weeks and with a slight amelioration towards the end of the follow up (Fig. 2B, group 7). In contrast, the joint swelling caused by Δ*dbpAB* (group 8) was mild and late onset emerging only at 10 weeks of the infection and showing statistically significant difference from the uninfected control at that time point (P ≤ 0.05).

On histological evaluation, findings in the joints at 15 weeks of Δ*dbpAB*/*dbpAB* infected mice showed thickening of the synovial membrane with proliferation of synovial lining cells, fibroblast and capillary proliferation as well as a mild chronic inflammation containing mainly lymphocytes (Fig. 2D). In addition, the articular cartilage surface showed mild degenerative changes. The findings in the joints of Δ*dbpAB* mice were minor and showed minimal thickening of the synovium consisting mainly of synovial fibroblasts, while no inflammatory cells, capillary proliferation or articular cartilage surface damage were seen (Fig. 2E).

These results indicate that Δ*dbpAB*/*dbpAB* causes a clear joint swelling and histologically evident arthritic lesions, while Δ*dbpAB* induces late onset swelling and only minor arthritis.

### Progression of the long-term infection

**Fig 3:**
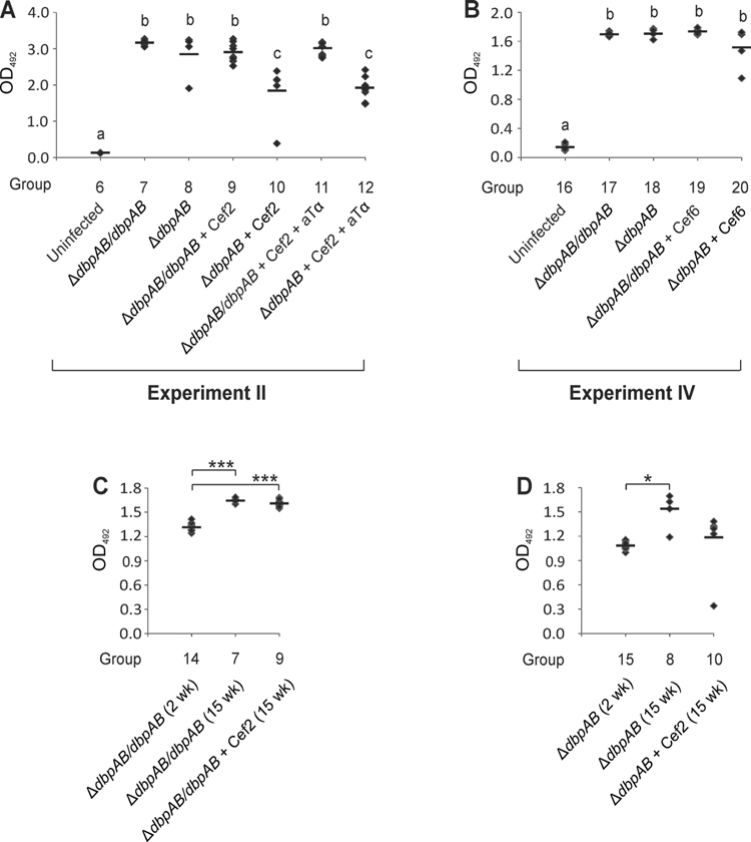
IgG antibody levels in mouse serum samples. Antibody levels were measured using enzyme immunoassays with whole *B*. *burgdorferi* lysate as antigen. In panel A, the results are from Experiment II, in panel B from Experiment IV, and in panels C and D combined from Experiments II and III. The results in each panel are obtained from an individual analysis. Each symbol represents the result of one animal. Results are expressed as OD492 values and all samples were analysed in duplicate. The line indicates the mean of each group. Groups with the same letter do not differ at 5% level of probability (Tukey’s HSD test, panels A and B). * P ≤ 0.05, *** P ≤ 0.001.

In experiment II, *B*. *burgdorferi* culture of ear biopsy samples taken at 6 and 9 weeks of the infection demonstrated that all Δ*dbpAB*/*dbpAB* and three out of four Δ*dbpAB* (transient infection in one mouse) infected mice developed disseminated infection (Table 2, groups 7 and 8). Serology using the whole cell antigen and performed on post mortem serum samples indicated that all animals mounted a clear IgG response against both *B*. *burgdorferi* strains (Fig. 3A, groups 7 and 8). Antibodies against C6 peptide were significantly increased only in the samples of Δ*dbpAB*/*dbpAB* infected mice (S1A Fig., groups 7 and 8). Δ*dbpAB*/*dbpAB* infected mice developed significantly elevated antibody levels also against DbpA and B (S1B and C Fig., group 7).

**Table 2:** *B*. *burgdorferi* culture results of Experiment II at 6, 9 and 15 weeks of infection.

		Ear Culture		Culture at 15 wk		
Group	Strain/treatment	wk 6	wk 9	Ear	Bladder	Joint
6	Uninfected	0/2	0/2	0/2	0/2	0/2
7	Δ*dbpAB*/*dbpAB*	4/4	4/4	4/4	4/4	4/4
8	Δ*dbpAB*	3/4	3/4	3/4	3/4	3/4
9	Δ*dbpAB*/*dbpAB* + Cef2	0/8	0/8	0/8	0/8	0/8
10	Δ*dbpAB* + Cef2	0/8	0/8	0/8	0/8	0/8
11	Δ*dbpAB*/*dbpAB* + Cef2 + aTα	0/8	0/8	0/8	0/8	0/8
12	Δ*dbpAB* + Cef2 + aTα	0/8	0/8	0/8	0/8	0/8

*B*. *burgdorferi* culture of post mortem tissue samples demonstrated widespread infection with positive culture results in all analysed tissues of Δ*dbpAB*/*dbpAB* infected mice and in the tissues of three out of four Δ*dbpAB* infected animals (Table 2, groups 7 and 8). Amplification of *B*. *burgdorferi flaB* and *ospA* genes using DNA extracted from joint tissue samples of Δ*dbpAB*/*dbpAB* infected mice corroborated the presence of *B*. *burgdorferi* in the joints of all Δ*dbpAB*/*dbpAB* infected and untreated mice (Table 3, group 7). Sensitivity of PCR appeared to be lower than the sensitivity of culture since 75% (¾) of ear samples of Δ*dbpAB*/*dbpAB* infected mice that were all culture positive were positive in PCR. This was even more pronounced with the samples of ΔdbpAB infected animals with only one out of three culture positive ear samples and none of the three culture positive bladder samples were PCR positive (Table 3, group 8).

**Table 3:** *B*. *burgdorferi* PCR results of Experiment II at 15 weeks of infection.

		***flaB***			*ospA*		
Group	Strain/treatment	Ear	Bladder	Joint	Ear	Bladder	Joint
6	Uninfected	0/2	0/2	0/2	0/2	0/2	0/2
7	Δ*dbpAB*/*dbpAB*	3/4	1/3^a^	4/4	2/4	4/4	4/4
8	Δ*dbpAB*	0/4	0/4	2/4	1/4	0/4	3/4
9	Δ*dbpAB*/*dbpAB* + Cef2	0/8	0/8	5/8	0/8	0/8	8/8
10	Δ*dbpAB* + Cef2	0/8	0/8	0/8	0/8	0/8	0/8
11	Δ*dbpAB*/*dbpAB* + Cef2 + aTα	0/8	0/8	8/8	0/8	0/8	8/8
12	Δ*dbpAB* + Cef2 + aTα	0/8	0/8	0/8	0/8	0/8	0/8

a One sample not available for *flaB* PCR

**Fig 4:**
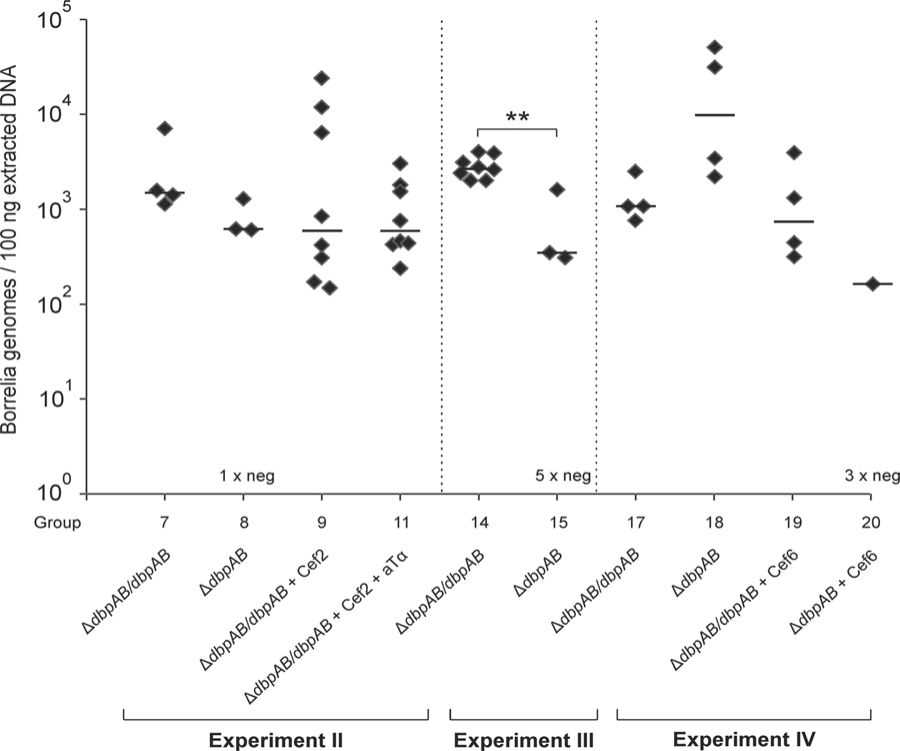
Bacterial load in joint samples. A 102 base pair fragment of *ospA* gene was amplified from the joint DNA samples. The minimal sensitivity of PCR was 40 bacterial cells. The results are expressed as the number of bacteria genomes per 100 ng of extracted DNA. Each symbol represents an individual animal and the samples were analysed in triplicate. In Experiment II, one joint sample of Δ*dbpAB* infected mice was qPCR negative at 15 weeks of infection, in Experiment III five joint samples of Δ*dbpAB* infected animals were qPCR negative at two weeks, and in Experiment IV three joint samples of Δ*dbpAB* infected and ceftriaxone treated (at six weeks of infection) mice were qPCR negative at 15 weeks. The line indicates the median of positive results in each group. “Cef” Ceftriaxone treatment, “aTα” anti-TNF-alpha treatment. ** P ≤ 0.01

The results of the quantitative *ospA* PCR showed that there was no statistically significant difference in the bacterial load between the *B*. *burgdorferi* PCR positive joint samples of Δ*dbpAB*/*dbpAB* and Δ*dbpAB* infected mice (Fig. 4, groups 7 and 8).

These results demonstrate that also the Δ*dbpAB* strain is able to persistently infect mice, and that the bacterial load in the joints of Δ*dbpAB* infected mice does not differ from the load of Δ*dbpAB*/*dbpAB* infected mice at 15 weeks of infection.

### Effect of ceftriaxone and anti-TNF-alpha treatments on dissemination and arthritis development

Ceftriaxone was administered twice a day for five days during the third week of infection in subgroups of Δ*dbpAB*/*dbpAB* and Δ*dbpAB* infected mice (Fig. 1, groups 9–12). Antibiotic susceptibility testing indicated that both strains were equally susceptible to ceftriaxone with MIC of 0.064 μg/ml. *B*. *burgdorferi* cultures of ear biopsy samples taken at 6 and 9 weeks of infection were negative in Δ*dbpAB*/*dbpAB* and Δ*dbpAB* infected mice suggesting clearance of spirochetemia (Table 2, groups 9 and 10). In parallel, the post mortem *B*. *burgdorferi* cultures of ear, bladder and joint tissues did not show growth, and, importantly, there was no measurable swelling in the tibiotarsal joints of the treated animals (Fig. 2B, groups 9 and 10). Immunosuppression of the mice with anti-TNF-alpha treatment (once a week during weeks 7 to 10) did not have any effect on the culture results or on the arthritis development (Table 2 and Fig. 2B, groups 11 and 12).

Taken together, the above results show that the presence in joints of cultivable *B*. *burgdorferi* with DbpA and B expression is a prerequisite for arthritis development in LB mouse model. Importantly, the results demonstrate that immunosuppression by anti-TNF-alpha treatment does not lead to activation of a possible latent infection and appearance of joint swelling in the antibiotic treated mice.

### Role of DbpA and B in persistence of *B*. *burgdorferi* DNA in mouse joints after ceftriaxone treatment at two weeks

As shown above, none of the infected and ceftriaxone treated animals were culture positive, and anti-TNF-alpha treatment had no effect on the culture positivity. However, *B*. *burgdorferi* DNA was PCR amplified from all joint tissues of Δ*dbpAB*/*dbpAB* infected and ceftriaxone (or ceftriaxone and anti-TNFalpha) treated mice 12 weeks after antibiotic treatment (Table 2, groups 9 and 11). Interestingly, all ear and bladder samples of the treated animals were PCR negative suggesting persistence of borrelial remnants specifically in joint tissue. Furthermore, none of the Δ*dbpAB* infected and treated mice retained borrelial DNA in the joints, or in ear and bladder tissues (Table 2, groups 10 and 12). The qPCR results of the joint tissues of treated and untreated Δ*dbpAB*/*dbpAB* infected mice demonstrated that ceftriaxone, or ceftriaxone and anti-TNF-alpha treatment, did not have a statistically significant effect on the *B*. *burgdorferi* DNA load in the tissues (Fig. 4, groups 7, 9 and 11).

Taken together, the presented results demonstrate the post-treatment persistence of borrelial DNA in mouse joints only when the animals are infected with a *B*. *burgdorferi* strain expressing DbpA and B adhesins.

### Analysis of the early dissemination of the infection

The finding that *B*. *burgdorferi* DNA is detected after antibiotic treatment solely in the joints of Δ*dbpAB*/*dbpAB* infected mice might be explained by a defect in the early dissemination and joint colonization of Δ*dbpAB* in mice. To investigate this, we analysed the culture and PCR positivity and bacterial load in tissues of Δ*dbpAB*/*dbpAB* and Δ*dbpAB* infected mice at 2 weeks of infection (Experiment III, groups 13–15). Of Δ*dbpAB*/*dbpAB* infected mice all analysed samples, except one ear, were culture positive and all joint samples contained borrelial DNA (Table 4, group 14). Of Δ*dbpAB* infected mice three out of eight joints contained cultivable *B*. *burgdorferi*, while all ear and bladder samples were culture negative (Table 4, group 15). In the PCR analyses, four joints of Δ*dbpAB* infected mice were borrelial DNA positive (one culture negative sample was PCR positive with both PCR methods). The bacterial load in the PCR positive joint samples was statistically significantly (P = 0.003) lower in Δ*dbpAB* infected mice than in Δ*dbpAB*/*dbpAB* infected mice (Fig. 4, groups 14 and 15). In conclusion, there clearly is a defect in the early dissemination and tissue colonization of the DbpA and B deficient strain. Interestingly however, while all ear and bladder samples of Δ*dbpAB* infected mice were *B*. *burgdorferi* negative, half of the animals were PCR and/or culture positive in the joints. This suggests that at least a part of Δ*dbpAB* infected mice that are treated with antibiotics at two weeks of infection harbour *B*. *burgdorferi* in the joints at that time point, and thus the lack of persisting DNA at 15 weeks of infection in the joints of these animals (Table 3, groups 10 and 12) cannot be explained by the absence of *B*. *burgdorferi* from the joints already before the treatment.

**Table 4:** *B*. *burgdorferi* culture and PCR results of Experiment III at two weeks of infection.

		Culture			PCR of joints		
Group	Strain/treatment	Ear	Bladder	Joint	*flaB*	*ospA*	Any method
13	Uninfected	0/4	0/4	0/4	0/4	0/4	0/4
14	Δ*dbpAB*/*dbpAB*	7/8	8/8	8/8	8/8	8/8	8/8
15	Δ*dbpAB*	0/8	0/8	3/8	3/8	3/8	4/8

### Effect of ceftriaxone and anti-TNF-alpha treatments on antibody responses

Ceftriaxone treatment, or ceftriaxone followed by anti-TNF-alpha treatment, did not abrogate the development of IgG antibodies against the whole cell antigen in Δ*dbpAB*/*dbpAB* infected mice (Fig. 3A, groups 7, 9 and 11). Indeed, there was a similar and statistically significant increase in the antibody levels from two to 15 weeks both in the untreated and treated mice (P < 0.001, Fig. 3C). In contrast, the antibody levels in the sera of Δ*dbpAB* infected and treated mice were lower than in the sera of untreated Δ*dbpAB* infected mice (Fig. 3A, groups 8, 10 and 12). Also, there was no statistically significant increase in the antibodies of Δ*dbpAB* infected and treated mice from two to 15 weeks, while the increase was significant in the untreated animals (P = 0.027, Fig. 3D).

C6 antibodies, and also anti-DbpA and anti-DbpB antibodies, in Δ*dbpAB*/*dbpAB* infected mice were statistically significantly lower in the treatment groups than in the untreated group (S1A, B and C Fig., groups 7, 9 and 11). No significant increase was detected in C6 antibodies of Δ*dbpAB* infected untreated mice when compared to the antibody levels of Δ*dbpAB* infected and treated animals (S1A Fig., groups 8, 10 and 12). As expected, no anti-DbpA and anti-DbpB antibodies were detected in the samples of Δ*dbpAB* infected mice (S1B and C Fig., groups 8, 10 and 12).

In conclusion, the finding that antibodies against whole cell antigen in the sera of Δ*dbpAB*/*dbpAB* infected and treated mice are similarly increased 12 weeks after the treatment as the antibodies in the sera of Δ*dbpAB*/*dbpAB* infected and untreated mice suggests the persistence of antigenic borrelial remnants in the antibiotic treated animals.

### Persistence *of B*. *burgdorferi* DNA in mouse joints after ceftriaxone treatment at six weeks

Experiment IV was performed to study the role DbpA and B in the persistence of borrelial DNA in mice treated with antibiotics at six weeks of the infection. This treatment time point was selected because at that point, also Δ*dbpAB* bacteria have disseminated and infected mice are spirochetemic as indicated by the ear biopsy sample cultures at six weeks of infection (Table 2, group 8), and by the culture results of Experiment I (Table 1). In this set up at 15 weeks of infection, a similar finding was evident as in Experiment II. All tested tissue samples of Δ*dbpAB*/*dbpAB* and Δ*dbpAB* infected and untreated mice were culture and PCR positive (Table 5, groups 17 and 18), while after ceftriaxone treatment, all samples of were culture negative (Table 5, groups 19 and 20). Importantly, all joint samples of Δ*dbpAB*/*dbpAB* infected and ceftriaxone treated animals retained borrelial DNA while none of the ear and bladder samples did (Table 5, group 19). The qPCR results of the joint samples revealed that there was no significant difference in the DNA load between the untreated and treated Δ*dbpAB*/*dbpAB* infected mice (Fig. 4, groups 17 and 19). Ceftriaxone treatment at six weeks time point abolished the joint swelling of Δ*dbpAB*/*dbpAB* infected mice (Fig. 2C, group 19). When the mice were infected with Δ*dbpAB* and treated at six weeks, one out of four joints samples contained *B*. *burgdorferi* DNA. The bacterial load in this sample was lower than in the joint samples of Δ*dbpAB*/*dbpAB* infected and treated mice (Fig. 4, groups 19 and 20). IgG levels against the whole cell *B*. *burgdorferi* antigen, C6 peptide, DbpA or DbpB in the sera of mice infected with Δ*dbpAB*/*dbpAB* or with Δ*dbpAB* were not affected by the ceftriaxone treatment at six week time point (Fig. 3B, S1D, E, F Fig., groups 17–20).

**Table 5:** *B*. *burgdorferi* culture and PCR results of Experiment IV at 15 weeks of infection.

		Culture at 15 wk			*OspA* PCR		
Group	Strain/treatment	Ear	Bladder	Joint	Ear	Bladder	Joint
16	Uninfected	0/4	0/4	ND	0/4	0/4	0/4
17	*ΔdbpAB*/*dbpAB*	4/4	4/4	ND	4/4	4/4	4/4
18	*ΔdbpAB*	4/4	4/4	4/4	4/4	4/4	4/4
19	Δ*dbpAB*/*dbpAB* + Cef6	0/4	0/4	0/4	0/4	0/4	4/4
20	Δ*dbpAB* + Cef6	0/4	0/4	0/4	0/4	0/4	1/4

ND Not determined

In conclusion, the results demonstrate that borrelial DNA persist specifically in the joint tissue of mice infected with DbpA and B expressing *B*. *burgdorferi* also when the mice are treated at a later time point of infection.

## Discussion

In the present study, we evaluated the role of DbpA and B adhesins in dissemination of LB, in the development of joint manifestations, and in bacterial persistence after ceftriaxone treatment in mice. The effect of immunosuppression by anti-TNF-alpha on the post-treatment progression of LB was evaluated. Specifically, immunosuppression was used to characterize the nature of the persisting material in antibiotic treated animals. The results show that, indeed, expression of both DbpA and B on *B*. *burgdorferi* is required for arthritis development. Also, post-treatment persistence of borrelial DNA in mouse joints was dependent on DbpA and B. Importantly, immunosuppression did not turn the joint tissue samples of the antibiotic treated animals to *B*. *burgdorferi* culture positive, thus suggesting that the persisting remnants in the joints of Δ*dbpAB*/*dbpAB* infected mice is DNA or DNA containing structures rather than live bacteria.

Mice were infected with previously characterized *B*. *burgdorferi* strains Δ*dbpAB* and Δ*dbpAB*/*dbpAB* [16]. The strains have been constructed in *B*. *burgdorferi* B31 5A13 background and are genetically identical except for the difference in the ability to express DbpA and B adhesins. The strains have the same plasmid content since both have lost plasmids cp9, lp5, lp21, lp28-4, lp25, and lp56 [16]. The positive C6 peptide antibody results (S1A and D Fig.) in most of Δ*dbpAB*/*dbpAB* and Δ*dbpAB* infected mice indicate that both strains retain the plasmid lp28-1 which is associated with an arthritic phenotype of *B*. *burgdorferi* [27]. Both strains are infectious in immunocompetent BALB/c mice, but the ID50 value of Δ*dbpAB* is 10^4^ times higher than the ID50 value of Δ*dbpAB*/*dbpAB*. However, the use of 10^6^ ΔdbpAB bacteria to infect BALB/c mice leads to joint infection [16]. Because of this, the C3H/HeN mice in the present study were infected with 10^6^ bacteria, which ensured that also Δ*dbpAB* inoculated mice developed disseminated infection.

Although Lyme arthritis is probably the most studied manifestation of LB, molecular mechanisms targeting *B*. *burgdorferi* to joint tissues and molecules responsible for the induction of arthritis are poorly understood. LB mouse model with inbred arthritis prone C3H mice is a widely used model system. Using this model it has been shown that several borrelial surface molecules, like basic membrane proteins A and B (BmpA and B) [28], and a recently discovered outer membrane protein BBA57 [29] participate in the genesis of murine Lyme arthritis suggesting that it is a multifactorial process. In addition, the role of DbpA has been studied in the context of joint colonization and arthritogenicity [21, 22]. The results by Fortune and others show that a knock out strain without DbpA and B expression does not infect mice at all, and that the expression of DbpA on *B*. *burgdorferi* was sufficient to restore infectivity and joint colonization. In contrast, the results of Lin and co-workers suggest that also the *dbpA/B* knock out strain is infectious in mice. They further show that the knock out strain expressing DbpA of *B*. *burgdorferi* colonizes tibiotarsal joint more than the knock out strain, and that the histologically evaluated joint inflammation score is higher in mice infected with this strain. Our results concerning the infectivity of the *dbpA/B* knock out strain are in line with the results by Lin and others, since also the strain used by us colonizes several mouse tissues including the tibiotarsal joint. In fact, our qPCR results of joint samples at week 15 indicate that the bacterial load does not differ between Δ*dbpAB*/*dbpAB* and Δ*dbpAB* infected mice. Also, antibodies against the whole cell antigen were similarly increased in mice infected with the two different strains. In general, our observations are in line with the results of Imai and co-workers who demonstrated that the early dissemination defect of *dbpA/B* deficient *B*. *burgdorferi* is abolished during the later stages of the infection [30].

In the present study, the arthritogenicity of *B*. *burgdorferi* strains in mice was evaluated primarily by measuring the diameter of the tibiotarsal joints. Using this approach it was evident that *B*. *burgdorferi* strains expressing either DbpA or B alone are not arthritogenic. Clearly, both DbpA and B are needed for full arthritis development since the joint diameter of Δ*dbpAB* infected mice remained at the background level until week 9 and showed slight increase only during weeks 10 to 15. The inflammation was evident also in the histological evaluation of joints of Δ*dbpAB*/*dbpAB* infected mice. The reason for the somewhat discrepant results between us and the studies by Fortune et al. and Lin et al. could be the use of different *B*. *burgdorferi* strains, in which the *dbpAB* deletion was generated, and the different sources of the *dbpA* and *B* genes used to construct the DbpA and B expressing strains.

It is becoming increasingly clear that in *B*. *burgdorferi* infected and antibiotic treated mice some sort of bacterial remnants may persist [5, 8, 9, 24, 31]. On the other hand, Liang and others have shown, using decorin knockout mice, that DbpA expressing *B*. *burgdorferi* are protected against mature immune response in foci with high decorin expression, like the joint tissue [23]. In the present study, we tested the hypothesis that the same niche is able to protect *B*. *burgdorferi* against antibiotic treatment. The results show that, indeed, only bacteria that express DbpA and B adhesins uniformly persist after ceftriaxone treatment (either at two or six weeks of infection) since borrelial DNA was detected exclusively in all of the joint samples of Δ*dbpAB*/*dbpAB* infected mice, while all other tissues were PCR negative. On the other hand, we could not culture Δ*dbpAB*/*dbpAB* (or Δ*dbpAB*) bacteria after ceftriaxone treatment from any of the tested samples, not even in the case of anti-TNF-alpha treatment induced immunosuppression. The rationale for using anti-TNF-alpha immunosuppression in two groups of antibiotic treated mice was that we have previously shown that *B*. *burgdorferi* infected C3H/HeN mice treated with ceftriaxone once a day for five days became *B*. *burgdorferi* culture positive after anti-TNF-alpha treatment [8]. However, in the present study with two daily doses of ceftriaxone, anti-TNF-alpha treatment did not reactivate the infection. Thus, when the antibiotic treatment is frequent enough DNA positivity of the joint tissue samples of Δ*dbpAB*/*dbpAB* infected mice rather suggests persistence of noncultivable borrelial remnants than an on-going infection. On the other hand, the persistence of antigenic remnants is supported by the similarly increased antibody levels against the whole *B*. *burgdorferi* antigen at 15 weeks of infection in treated (two or six weeks) and non-treated mice.

Bockenstedt and co-workers have elegantly shown that immunogenic antigens persist in mouse patellae after antibiotic treatment in a murine LB model [9]. They prepared homogenates from patellae of infected and antibiotic treated mice, and used the extract to immunize naïve mice. Finally, they showed that in the sera of the immunized mice there were antibodies that recognized *B*. *burgdorferi* proteins on Western blot. From this, they draw the conclusion that there are persisting borrelial antigens in the joints of the antibiotic treated mice. Inspired by this, we also tried to demonstrate the presence of immunogenic *B*. *burgdorferi* antigens in the PCR positive tibiotarsal joints of infected and untreated, or infected and ceftriaxone treated (at two weeks) mice (Salo et al, unpublished results). Tibiotarsal joint samples were homogenized and proteins extracted using a commercial protein extraction kit. Naïve C3H mice were immunized using a mixture of the protein extract (100 μg) and an adjuvant (TiterMax® Gold Adjuvant, Sigma-Aldrich). The mice were booster immunized two weeks later with 50 μg of the extract. Sera were collected two weeks after the second immunization and used to probe *B*. *burgdorferi* lysate on Western blots. One to four bands were detected in the Western blot analysis using any of the post-immunization sera, while, however, none of them appeared to be *B*. *burgdorferi* specific, since all of the bands were also detected on a blot that was probed with the serum of the adjuvant only immunized animal. The reason for the discrepant results of our experiments v. the results of Bockenstedt and others’ is unclear. However, the mouse strain used by us was different, and we did not prepare the patellae of the mice, but instead used extracts of the whole tibiotarsal joints in the mice. Thus, this experiment did not clarify the nature of the persisting material in the mouse joints, and therefore the data of the experiment are not shown.

In conclusion, the results of the present paper show that both decorin binding proteins A and B of *B*. *burgdorferi* are needed for early and prominent arthritis development in mice although also *B*. *burgdorferi* strains that express DbpA or B alone, or the strain that is DbpA/B deficient, are able to colonize mouse joints. The progression of the joint manifestations in Δ*dbpAB*/*dbpAB* infected mice is biphasic with peaks at 4 and 9–11 weeks of infection, and with histologically evident arthritis at 15 weeks of infection. The most important finding of the present study is the absence of post treatment borrelial DNA persistence in the joints of mice infected with DbpA/B deficient *B*. *burgdorferi*, while in the mice infected DbpA and B expressing *B*. *burgdorferi*, all joint samples were borrelial DNA positive up to 12 weeks after the treatment. One obvious explanation for this phenomenon is that DbpA and B assist the bacteria in invasion to decorin rich foci in mouse joints, which in turn allows evasion of antibiotic treatment and leads to post-treatment persistence of bacterial remnants in mouse joints. Based on the results of our anti-TNF-alpha immunosuppression experiments, the nature of the persisting material in the antibiotic treated mice appears to be non-cultivable bacterial remnants. The finding that only *B*. *burgdorferi* with a particular set of adhesins can form deposits of persisting remnants after treatment is thought provoking. We and others have shown that DbpA and B molecules of different *B*. *burgdorferi* sensu lato genospecies have different abilities to mediate binding to decorin and to decorin expressing cells [22, 26, 32]. Therefore, we will next focus on evaluating the contribution of DbpA and B of different *B*. *burgdorferi* sensu lato genospecies to dissemination of the infection, to arthritis development and to the post treatment persistence potential.

## Supporting Information

S1 Fig IgG antibodies against C6, DbpA and DbpB in mouse serum samples. Antibody levels were measured using enzyme immunoassays with C6 peptide (A and D), DbpA (B and E) and DbpB (C and F) as antigens. Each symbol represents the result of an individual animal. Results are expressed as OD_492_ values and all samples were analysed in duplicate. The line indicates the mean of each group. Groups with same letter do not differ at 5% level of probability (Tukey’s HSD test).
(TIF)
