# Supplementary figures and images for: Decorin Binding Proteins of Borrelia burgdorferi Promote Arthritis Development and Joint Specific Post-Treatment DNA Persistence in Mice
Source: PLoS One. 2015 Mar 27;10(3):e0121512. doi: 10.1371/journal.pone.0121512 (PMC4376631; doi:10.1371/journal.pone.0121512)

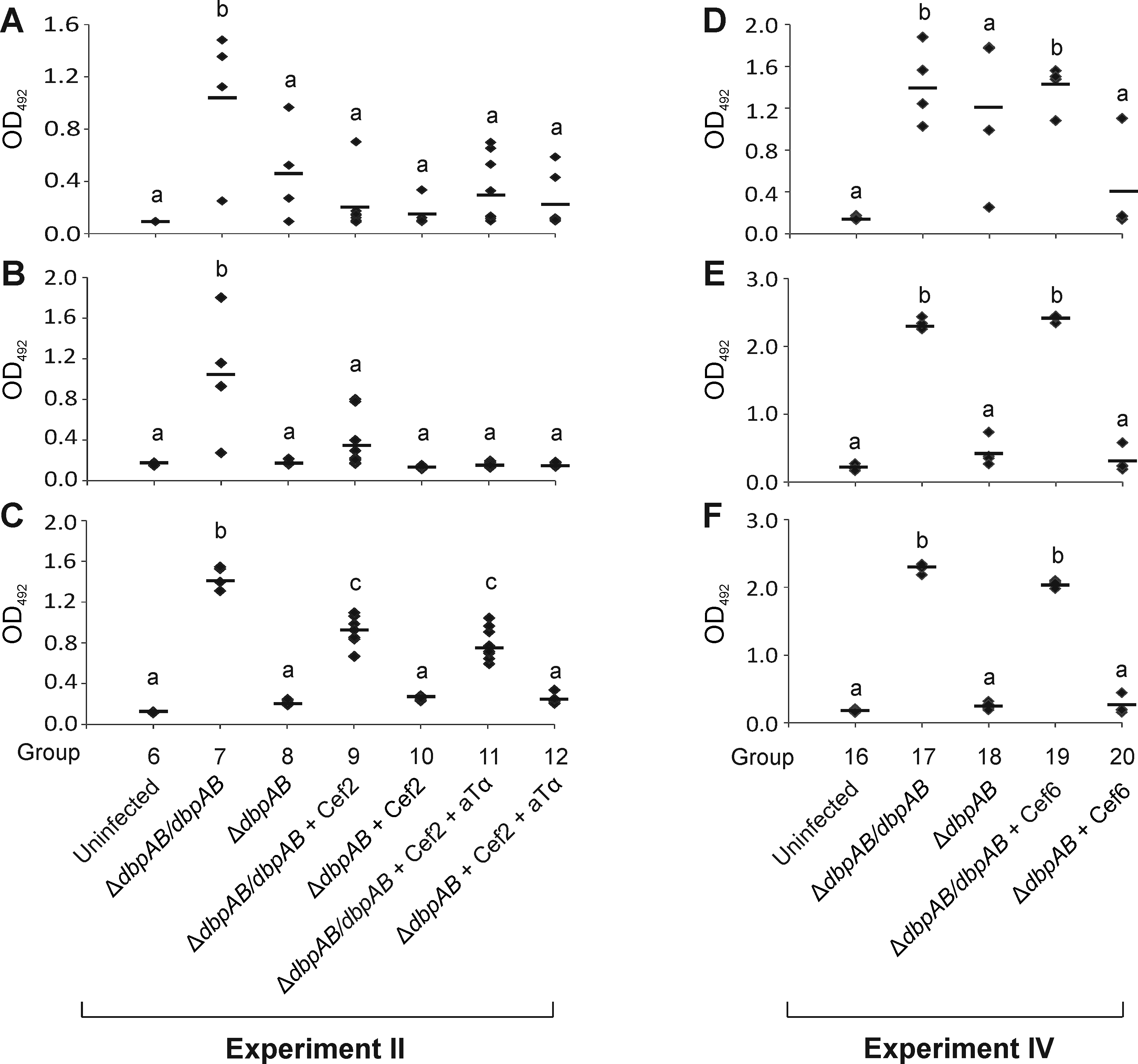

Supplement: S1 Fig — Antibody levels were measured using enzyme immunoassays with C6 peptide (A and D), DbpA (B and E) and DbpB (C and F) as antigens. Each symbol represents the result of an individual animal. Results are expressed as OD492 values and all samples were analysed in duplicate. The line indicates the mean of each group. Groups with same letter do not differ at 5% level of probability (Tukey’s HSD test). (TIF) [file pone.0121512.s001.tif]
